# Enhancement of IL-6 Production Induced by SARS-CoV-2 Nucleocapsid Protein and Bangladeshi COVID-19 Patients’ Sera

**DOI:** 10.3390/v15102018

**Published:** 2023-09-28

**Authors:** Abu Hasan, Rummana Rahim, Emi E. Nakayama, Kazuko Uno, Nazmul Hasan, Mizanur Rahman, Tatsuo Shioda

**Affiliations:** 1 Evercare Hospital Dhaka, Plot-81, Block-E, Bashundhara R/A, Dhaka 1229, Bangladesh; rasel.hasan@evercarebd.com (A.H.); rummana.rahim@evercarebd.com (R.R.); nazmul1.hasan@evercarebd.com (N.H.); 2 Research Institute for Microbial Diseases, Osaka University, Suita 565-0781, Japan; emien@biken.osaka-u.ac.jp; 3 IFN & Host-Defense Research Laboratory, Louis Pasteur Center for Medical Research, Kyoto 606-8225, Japan; kazukouno@louis-pasteur.or.jp

**Keywords:** COVID-19, SARS-CoV-2, IL-6, N protein, anti-N antibody, nucleocapsid, Ep9 epitope

## Abstract

Coronavirus disease 2019 (COVID-19) is a respiratory tract infection caused by severe acute respiratory syndrome coronavirus 2 that can have detrimental effects on multiple organs and accelerate patient mortality. This study, which encompassed 130 confirmed COVID-19 patients who were assessed at three different time points (i.e., 3, 7, and 12 days) after the onset of symptoms, investigated interleukin-6 (IL-6) enhancement induced by a viral nucleocapsid (N) protein from a myeloid cell line. Disease severity was categorized as mild, moderate, or severe. The severe cases were characterized as having significant elevations in serum IL-6, C-reactive protein, D-dimer, ferritin, creatinine, leukocytes, and neutrophil-to-lymphocyte ratio and decreased hemoglobin, hematocrit, and albumin levels compared with mild and moderate cases. To evaluate IL-6-inducing activity, heat-inactivated sera from these patients were incubated with and without the N protein. The findings showed a progressive increase in IL-6 production in severe cases upon N protein stimulation. There was a strong correlation between anti-N antibodies and levels of IL-6 secreted by myeloid cells in the presence of N protein and sera, indicating the crucial role that the anti-N antibody plays in inducing IL-6 production. Uncontrolled IL-6 production played a pivotal role in disease pathogenesis, exacerbating both disease severity and mortality. Efficiently targeting the N protein could potentially be employed as a therapeutic strategy for regulating the immune response and alleviating inflammation in severe cases.

## 1. Introduction

The outbreak of coronavirus disease 2019 (COVID-19) that occurred in Wuhan, China, in December 2019 became a serious threat to public health around the globe [1]. COVID-19 is caused by severe acute respiratory syndrome coronavirus 2 (SARS-CoV-2), which belongs to the Coronaviridae family [2]. The genome contains open reading frames (ORFs) that code for structural, non-structural, and accessory proteins [3,4,5,6,7,8,9]. The spike (S), nucleocapsid (N), membrane (M), and envelope (E) proteins are structural proteins that form virus particles [4]. COVID-19 is a respiratory tract infection with the common clinical symptoms of fever, cough, and fatigue; in severe cases, the disease may also present with dyspnea and systemic manifestations. Multi-organ involvement, including the gastrointestinal tract; liver; kidney; and cardiovascular, neurological, hematopoietic, and immune systems, has also been reported [10,11,12,13,14,15]. Alterations in hematological and biochemical parameters are also common in severe cases, exemplified by lymphopenia, hypoalbuminemia, and higher levels of alanine aminotransferase (ALT), lactate dehydrogenase, C-reactive protein, ferritin, and D-dimer [16]. During SARS-CoV-2 infection, host cells produce numerous cytokines and chemokines to initiate inflammatory responses and to mediate innate immune responses [17,18]. In COVID-19 cases, several cytokines are associated with rapid disease progression and a higher complication rate, and interleukin-6 (IL-6) is the most notable cytokine among them [19,20,21,22,23,24,25]. IL-6 is a pro-inflammatory cytokine that regulates cell proliferation, differentiation, and apoptosis and has been implicated in the pathogenesis of COVID-19 [26]. It is released by a variety of cells, including T cells, B cells, macrophages, and endothelial cells in response to bacterial, viral, and other microbial infections.

Since high levels of IL-6 in blood can indicate inflammation, infection, cardiovascular diseases, or autoimmune disorders [27,28,29,30], IL-6 can also be used as a biomarker for disease progression in COVID-19 patients [31]. Continuous exposure to elevated IL-6 levels stimulates the immune system, impairs cytolytic function, and can result in multiple organ failure [32]. An increase in IL-6 levels in the early stage of infection indicates potential deterioration in COVID-19 patients. As a result, the patients require vigilant monitoring and control measures for both infection and inflammation to prevent further deterioration [33]. An inflammatory storm caused by IL-6 that leads to the sudden deterioration of a patient can potentially be minimized by suppressing IL-6 function. In fact, tocilizumab, a monoclonal antibody against IL-6 receptors, has been shown to be effective in treating COVID-19 [34,35,36]. Consequently, serum IL-6 levels should be monitored regularly after admission to predict disease severity and to administer immune-modulating therapy when required [37].

Shimizu et al. described a method for evaluating the antibody-dependent enhancement (ADE) of infection using live SARS-CoV-2 in an induced pluripotent stem (iPS)-cell-derived myeloid cell line (clone #35) expressing angiotensin-converting enzyme 2 (ACE2), which acts as a receptor for the SARS-CoV-2 virus and allows it to infect the cell, and transmembrane serine protease 2 (TMPRSS2), which is involved in the entry and spread of coronaviruses. They also examined the potential of sera from COVID-19 patients to enhance and augment IL-6 production [38]. Using 1000 ng/mL of recombinant spike (S) and nucleocapsid (N) proteins from SARS-CoV-2, Karwaciak et al. reported that both proteins induced IL-6 in monocytes and macrophages [39]. Zhang et al. showed the requirement for the 80 amino acid residues at the C terminus of the N protein of the SARS virus, a closely related coronavirus that emerged in 2003, for IL-6 production using the NF-kB transcription factor [40]. Pan et al. reported that the C-terminal domain of the SARS-CoV-2 N protein interacts with the nucleotide oligomerization domain (NOD), leucine-rich repeat (LRR), and pyrin domain-containing protein 3 (NLRP3) to activate inflammasome and the NF-kB pathway [41]. We previously showed that the SARS-CoV-2 N protein has the potential to induce IL-6 production in a parental myelomonocytic leukemia (K-ML2) cell line of clone #35 [38], lacking ACE2 and TMPRSS2, without SARS-CoV-2 replication [42]. The SARS-CoV-2 N protein induced IL-6 production more potently than the SARS-CoV-2 S protein. In addition, anti-N protein monoclonal antibodies enhanced the IL-6 production that was induced by the N protein. Furthermore, in K-ML2 cells cultured with SARS-CoV-2 N protein, patient sera obtained from severe cases induced more IL-6 than those from mild cases. In the present study, COVID-19 samples were collected at three different time points of the disease course, and sera were used with the N protein in K-ML2 cell culture to clarify the enhancement of IL-6 production. Here, we show that sera collected from severe cases 2–3 days after the onset of symptoms have the potential to further induce IL-6 through anti-N antibodies in the presence of the N protein.

## 2. Materials and Methods

### 2.1. Ethical Approval

Approval for this SARS-CoV-2 study was obtained from the Research and Ethical Practice Committee of Evercare Hospital Dhaka (approval number ERC 33/2022-01); the Research Ethics Committee of the Research Institute for Microbial Diseases, Osaka University, Japan (No. 2021-3); and the Louis Pasteur Center for Medical Research, Kyoto, Japan (LPC29). De-identified serum samples stored with different codes at −80 °C were used in this study. This study was exempt from the requirement for obtaining the participants’ consent, as only leftover specimens were used after anonymization.

### 2.2. Study Population and Data Collection

The aims of the present study were (1) to re-evaluate the enhancement of N protein-mediated IL-6 production from myeloid cells induced by patient sera reported in our previous study [42] and (2) to clarify the roles of the anti-N antibody in this phenomenon. Patients with clinical suspicion of SARS-CoV-2 infection who visited Evercare Hospital Dhaka from 25 December 2021 to 21 September 2022 were considered as candidates in this study, irrespective of their ages. Patients who underwent additional biochemical or hematological investigations remained candidates, while those who did not undergo any investigations except for COVID-19 RT-PCR tests were excluded. Finally, those patients who were COVID-19 RT-PCR-negative were excluded. Blood samples that remained after samples were submitted for the screening of hematological and biochemical parameters were collected for this study. The clinical information of the patients was obtained from the hospital information system of Evercare Hospital Dhaka, Bangladesh. We included a total of 130 COVID-19 RT-PCR-confirmed cases (outpatients, *n* = 39; inpatients, *n* = 91) and divided them into three groups (mild, moderate, and severe) according to disease severity, as defined in the hospital COVID-19 taskforce management guidelines. We did not perform any sample size calculations before including these patients in the study, as the effect size and statistical power may differ between Japan and Bangladesh.

Disease severity was not determined at the time of the patients’ first visits; rather, it was mentioned in their case sheets at the time of discharge from the hospital. The “mild” category comprised patients with laboratory-confirmed COVID-19 with one or more of the following COVID-19 symptoms: fever, cough, runny nose, fatigue, headache, nausea, vomiting, diarrhea, chest pain, abdominal pain, and loss of taste or smell. However, this category excluded patients with shortness of breath and dyspnea on exertion and those who showed abnormal radiological findings. The “moderate” category comprised patients with laboratory-confirmed COVID-19 who presented with pneumonia, blood oxygen saturation >93% on room air, and/or who required minimal oxygen support. The “severe” category comprised patients who developed COVID-19 pneumonia as evidenced by radiological imaging findings and required hospitalization, as well as patients who developed dyspnea, had a respiratory frequency of ≥30 breaths/min, had blood oxygen saturation levels of ≤93% on room air, had lung infiltrates in >50%, or required mechanical ventilation and/or ICU support.

In cases where COVID-19 was confirmed via RT-PCR, we collected information on routine blood biomarkers (i.e., white blood cell (WBC), neutrophil, lymphocyte, and platelet counts and hemoglobin and hematocrit levels), levels of C-reactive protein (c), ferritin, D-dimer, procalcitonin, creatinine, ALT, aspartate aminotransferase (AST), albumin, high-sensitivity troponin I (hs-TnI), and N-terminal pro-b-type natriuretic peptide (NT-proBNP). Clinical presentations including fever, cough, runny nose, shortness of breath, nausea or vomiting, diarrhea, fatigue, headache, chest pain, abdominal pain, myalgia, or arthralgia were noted based on the information in the hospital information system. Patient characteristics, such as age, sex, duration of hospital stay, treatment, and clinical outcomes were also noted.

### 2.3. Molecular Testing for SARS-CoV-2 Detection

Nasopharyngeal swabs were collected from suspected COVID-19 patients by trained medical personnel. The collected swabs were transferred immediately to viral transport medium (VTM) and sent to the Molecular Diagnostics Lab of Evercare Hospital Dhaka for further testing. For qualitative RT-PCR analysis, samples containing VTM were extracted automatically using a KingFisher Flex-Automated Extraction Analyzer (Thermo Fisher Scientific, Waltham, MA, USA) and a MagMAX Viral/Pathogen Kit (Applied Biosystems by Thermo Fisher Scientific, Waltham, MA, USA). PCR was performed using a Novel Coronavirus (2019-nCoV) Nucleic Acid Diagnostic Kit (Sansure Biotech, Changsha, China), according to the manufacturer’s instructions. This assay utilizes double-target genes, 2019-nCoV ORF1ab, and a specific conserved sequence of the N protein-coding gene. The PCR detection system also uses a positive internal control (RNase P) to assess the presence of PCR inhibitors in a test specimen and to avoid false negative results. The results were considered valid when the threshold cycle (Ct) value obtained for the reference gene was <40. The result was considered positive when the Ct values of all three targets were <40 with a typical S-shaped curve and negative when there was no Ct value or the Ct was ≥40.

### 2.4. Hematological Parameter Detection

Complete blood counts, including WBC, neutrophil, lymphocyte, hemoglobin, and platelet counts, as well as hematocrit measurements, were performed using an automated hematology analyzer (XN 2000, Sysmex Corporation, Kobe, Japan). The neutrophil-to-lymphocyte ratio (NLR) was calculated by dividing the number of neutrophils by the number of lymphocytes. The fibrin degradation product D-dimer, a marker that is commonly used to test and evaluate clot formation, was measured using an automated hemostasis benchtop analyzer (CS-1600, Sysmex Corporation, Kobe, Japan).

### 2.5. Biochemical Parameter Detection

Inflammation was detected using the CRP test using an automated hemostasis benchtop analyzer (BN ProSpec System, Siemens, Berlin, Germany) and a clinical chemistry analyzer (DxC 700AU, Beckman Coulter, Brea, CA, USA). The ferritin test is used to determine how much iron a human body can store. ALT, AST, and albumin are liver function tests that are used to screen, diagnose, or monitor liver problems. The highly sensitive Troponin I (hs-TnI) blood test is used to evaluate the condition of the heart, as Troponin-I is released when the heart muscles are damaged. The N-terminal pro-b-type natriuretic peptide (NT-proBNP) test is typically used to diagnose or exclude heart failure and also to assess the severity of the condition. The procalcitonin test is performed if a patient has a high risk of developing sepsis or if they have sepsis from a bacterial infection. The ferritin, ALT, AST, albumin, hs-TnI, NT-proBNP, and procalcitonin tests were all performed using an automated laboratory analyzer (Dimension EXL 200, Siemens, Berlin, Germany) and an automated immunoassay system (ADVIA Centaur XP, Siemens, Berlin, Germany).

### 2.6. K-ML2 Cell Culture and Induction with SARS-CoV-2 N Protein

K-ML2 cells are iPS cell-derived myeloid cells that express genetically engineered granulocyte–macrophage colony-stimulating factor and macrophage colony-stimulating factor [38], and they can be easily maintained and expanded as an established cell line. K-ML2 cells were maintained in minimum essential medium (MEM) supplemented with 10% fetal calf serum. Two microliters of COVID-19 heat-inactivated serum was added to each well of a 48-well plate. SARS-CoV-2 N protein (Source: Sino Biological Inc., Bejing, China, Cat: 40588-V07E, Lot: LC14AU1211) was then also added to each well at a concentration of 31.2 ng per 100 µL. Aliquots of K-ML2 cells (4 × 10^5^) suspended in 100 µL medium were then added to each well and incubated at 37 °C for 4 h. Following incubation, 500 µL of fresh medium was added to each well. Culture supernatants were harvested after 2 days, and the level of IL-6 was measured using an enzyme-linked immunosorbent assay (ELISA) kit, as described below. The K-ML2 cell line, with or without the N protein and without the addition of patient sera, was used as a control.

### 2.7. IL-6 Measurement from Myeloid Cell Culture and Directly from Untreated Serum

Levels of IL-6 from harvested K-ML2 culture supernatants were measured using an ELISA MAX Deluxe Set Human IL-6 kit according to the manufacturer’s instructions (BioLegend, San Diego, CA, USA). Briefly, 100 µL of 5× diluted antibody-captured solution was added to each well of a new ELISA plate and incubated at 4 °C overnight. After incubation, 200 µL of assay diluent was added to each well, and the wells were sealed and incubated for 1 h. After washing with 0.05% Tween 20 in phosphate-buffered saline (PBS-T), 100 µL of 1% diluted heat-inactivated patient serum and serially diluted standards were added to appropriate wells and incubated at room temperature (RT) for 2 h. After washing with PBS-T, 100 µL of 200× diluted detection antibody was added, and the samples were incubated at RT for 1 h. Then, 100 µL of 1000× diluted Avidin-HRP solution was added after washing, and the samples were incubated on a plate for 30 min at RT. A tetramethylbenzidine (TMB) substrate solution was used for colorimetric detection, and the optical density was measured at 450 nm and 570 nm using a multigrading microplate reader (SH-9500Lab, Corona Electric Co., Ltd., Hitachinaka-shi, Japan). A standard curve was generated using serially diluted standards, and the concentration of each sample was measured (450–570 nm) and recorded using the standard curve.

Levels of IL-6 were also measured directly from untreated serum samples of COVID-19 patients according to the manufacturer’s instructions. A human cytokine assay kit (Bio-plex Pro™, Bio-Rad, Hercules, CA, USA) was used for IL-6 measurement with an automated immunoassay multiplex array system (Luminex Bio-Plex 200, Bio-Rad, Hercules, CA, USA).

### 2.8. In-House ELISA Measurement of Anti-N and Anti-N Protein Epitope (Ep9) Antibodies from Heat-Inactivated Serum

Ninety-six-well, flat-bottomed microplates were coated with 100 ng/well of N protein (40588-V08B, Sino Biological Inc., Eschborn, Germany) or Ep9 epitope (ANNAAIVLQLPQGTTLPKGFY) [43] in 50 μL of carbonate–bicarbonate buffer (C-3041, Sigma, St. Louis, MO, USA) and incubated at 4 °C overnight. After washing with PBS-T, the wells were blocked with 200 μL of 25% Block Ace solution for 1 h at RT. Microplates were washed after incubation, 50 µL of patient serum diluted 100× with PBS-T was then added to each well, and the plates were incubated for 1 h at RT. After washing with PBS-T, 50 μL of the secondary antibody solution consisting of peroxidase-conjugated AffiniPure alpaca anti-human IgG (H+L) (609-035-213, Jackson ImmunoResearch, Pennsylvania, PA, USA) or peroxidase-labeled goat anti-mouse IgG (H+L) (5220–0341, CeraCare, Milford, MA, USA) was added to each well and incubated for 1 h at RT. A TMB substrate kit (34021, Thermo Fisher Scientific, Waltham, MA, USA) was used for colorimetric detection, and the optical density at 450 nm was measured using a multigrading microplate reader (SH-9500Lab, Corona, Hitachinaka, Ibaraki, Japan).

## 3. Data Processing and Analysis

The collected data were processed and analyzed using the SPSS software package (version 29) and GraphPad Prism 10 (Boston, MA, USA). We used a non-parametric method for statistical evaluation since the values did not follow a normal distribution. The statistical significance of differences was estimated using the non-parametric Kruskal–Wallis test, Pearson’s chi-square test, Fisher’s exact test, and Spearman’s test. Wilcoxon’s signed rank test was applied for repeated measures of longitudinal analysis. A *p*-value of <0.05 was considered statistically significant. Corrections for multiple comparisons were not performed.

## 4. Results

### 4.1. Demographics and Clinical Characteristics

A total of 130 patients with SARS-CoV-2 confirmed with PCR were included in the present study. Disease severity in the patients was retrospectively categorized as mild, moderate, or severe. Since older individuals (>65 years of age) are at greater risk of severe disease [44], we compared the age and sex distribution with disease severity. The median age of the study population was 57 years. More severe cases were observed in patients >70 years of age. Deaths occurred in severely ill patients with a median age of 78 years. Male predominance (60%) was observed in the present study, which is similar to observations in a previous study [45]. Fever was the predominant symptom among mild (90.9%), moderate (82.36%), and severe (100%) cases, followed by cough and fatigue. A marked shortness of breath was noted in severe cases (83.3%) (Table 1).

### 4.2. Vaccination Status and Clinical Outcome

Although all severe cases were vaccinated (information regarding manufacturer, type, and dose were not available), 61.1% of deaths occurred in this group, and the median hospital stay was 10 days. Hospital admission was required in 91.3% of moderate cases, which had a median hospital stay and vaccination rate of 5 days and 97.8%, respectively. No fatalities were recorded in the mild or moderate groups. More than half of mild cases (53%) were treated on an outpatient basis, and some were hospitalized because of anxiety related to COVID-19. Two mild cases had longer hospital stays (>8 days), as they wished to be monitored continuously until they fully recovered from COVID-19 (Table 1).

### 4.3. Laboratory Findings

We collected samples from each of patient with SARS-CoV-2 on Day 1, Day 5, and Day 10 of their hospital stay. Approximately 2–3 days after the onset of symptoms, the day of the first COVID-19 RT-PCR sampling was considered Day 1 for each severity category (mild, moderate, and severe). Day 5 and Day 10 cases were counted as the 5th and 10th days after being confirmed as being SARS-CoV-2-positive. We compared the biochemical and hematological parameters of all Day 1 samples for each severity category. For CRP, D-dimer, ferritin, creatinine, serum IL-6, WBC, and NLR, statistically significant trends toward higher titers were observed in more severe cases (*p* < 0.001, Kruskal–Wallis H-test). Similarly, AST, procalcitonin, hs-TnI, and NT-proBNP levels were also higher in more severe cases (*p* < 0.05, Kruskal–Wallis H-test). Hemoglobin, hematocrit, and albumin values were all significantly lower in the more severe cases (*p* < 0.001, Kruskal–Wallis H-test). These trends were further confirmed using post hoc pairwise comparisons between mild and moderate, moderate and severe, and mild and severe cases (Mann–Whitney U-test, Figure S1). No significant differences were observed in ALT and platelet values (Table 2 and Figure S1). These results were consistent with numerous previous studies [46,47,48,49,50,51,52,53,54,55,56,57,58] and confirmed that the patients were grouped appropriately.

### 4.4. Enhancement of IL-6 Production from Myeloid Cell Line Induced by COVID-19 Patients’ Sera at Different Time Points

**Figure 1:**
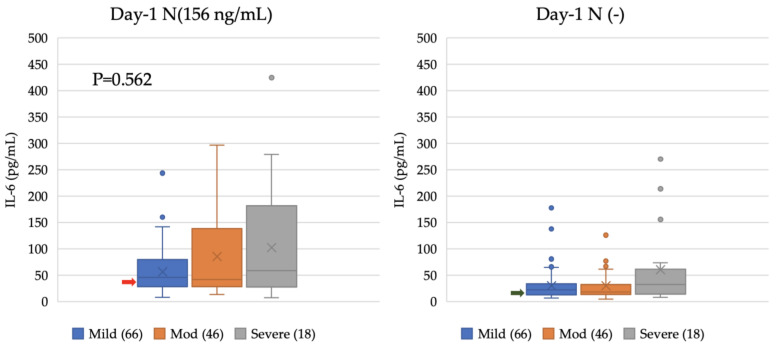
IL-6 induction with COVID-19 serum samples collected on Day 1. A total of 156 ng/mL of N protein was added to K-ML2 cells to enhance IL-6 production together with 1% patient serum. The blue, orange, and gray bars denote mild, moderate, and severe cases, respectively. The horizontal lines in the boxes, the boxes, and the vertical bars indicate the median, 25/75 percentiles, and 5/95 percentiles, respectively. Red and green arrows indicate IL-6 levels with N protein only and base levels of IL-6, respectively. “X” indicates the mean. N indicates the presence of N protein, and N(−) indicates the absence of N protein.

To evaluate the IL-6-inducing activity of the patients’ sera, as described previously [42], heat-inactivated serum samples collected from 130 (66 mild, 46 moderate, and 18 severe) COVID-19 patients at the time of diagnosis (Day 1) were incubated with and without the N protein to clarify the extent of IL-6 enhancement. Without patient sera, the IL-6 levels in the K-ML2 cell culture incubated with the N-protein only were 41.87 pg/mL compared with a basal IL-6 level of 18.30 pg/mL. When we added the patients’ sera, the median IL-6 concentration was higher in samples from severe cases (59.10 pg/mL, IQR; 29.28–152.11 pg/mL) than those in samples from mild (46.02 pg/mL, IQR; 28.73–77.65 pg/mL) and moderate cases (41.99 pg/mL, IQR; 28.25–137.58 pg/mL), although this difference was not significant (*p* = 0.562) when analyzed using the Kruskal–Wallis H-test (Figure 1). In the absence of the N protein, a small amount of patient sera also induced IL-6 production via K-ML2 cells.

Without the N protein, the average 95th percentile of the IL-6 levels in cell cultures with Day 1 sera was 126 pg/mL. We found that 4 (6%) out of 66 mild cases, 13 (28%) out of 46 moderate cases, and 5 (28%) out of 18 severe cases exceeded 126 pg/mL when the N protein was added to the culture (Table 3). A significant difference in the proportion of specimens with IL-6 levels over 126 pg/mL was observed in these three categories (*p* < 0.05, Fisher’s exact test), and a trend toward higher levels of IL-6 induction in more severe patients was clearly observed. These results corroborated our previous findings, which showed that IL-6 production by the K-ML2 cell line in response to the presence of the N protein was enhanced by the patients’ sera and that sera from moderate and severe cases enhanced IL-6 production more than sera from mild cases [42].

With N protein stimulation, 52 Day 5 samples comprising 9 mild, 28 moderate, and 15 severe cases showed median (IQR) IL-6 concentrations of 136.43 pg/mL (72.83–184.51 pg/mL), 70.06 pg/mL (38.80–188.25 pg/mL), and 80.78 pg/mL (42.95–202.99 pg/mL), respectively. Without N protein stimulation, the IL-6 concentrations were 43.67 pg/mL (21.87–61.51 pg/mL), 34.64 pg/mL (20.51–114.04 pg/mL), and 34.52 pg/mL (17.53–81.69 pg/mL), respectively (Figure S2). These results showed that IL-6 induction was enhanced after exposure to patient sera from all three COVID-19 severity categories.

For Day 10 samples, which were composed of 2 mild, 10 moderate, and 10 severe cases, the median (IQR) values obtained for IL-6 production were 109.76 pg/mL (100.33–119.19 pg/mL) for mild cases, 192.65 pg/mL (137.98–260.92 pg/mL) for moderate cases, and 109.82 pg/mL (83.16–240.06 pg/mL) for severe cases in the presence of the N protein. Without the N protein, the values were 77.53 pg/mL (66.08–88.98 pg/mL), 66.99 pg/mL (42.71–126.57 pg/mL), and 54.28 pg/mL (38.55–99.64 pg/mL), respectively (Figure S2). Therefore, Day 10 samples tended to induce more IL-6 than Day 1 and Day 5 samples in the presence of N protein stimulation. A similar, but weaker, tendency was also observed in the absence of N protein stimulation.

### 4.5. Longitudinal Analysis of Potency of IL-6 Induction via Patient Samples

**Figure 2:**
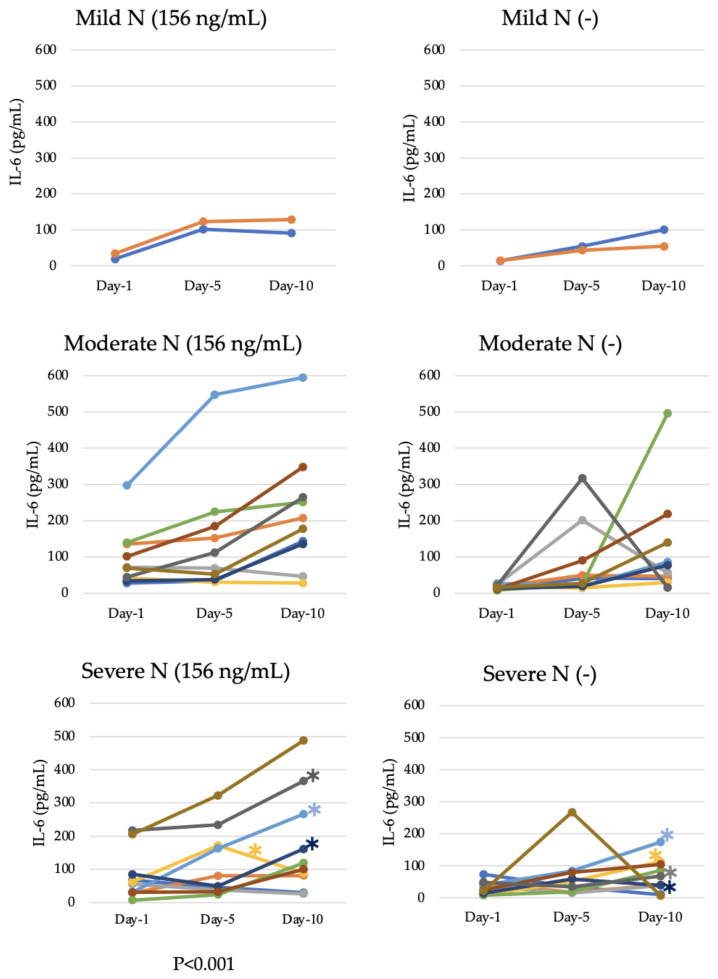
Longitudinal analysis of IL-6 enhancement induced by COVID-19 patient sera. IL-6 levels in K-ML2 cell culture with Day 1, Day 5, and Day 10 serum samples of patients were measured to observe N protein-induced enhancement. Each color denotes an individual case. Asterisks (*) indicate patients who died. Different colored lines indicate different patients. N indicates the presence of N protein, and N(−) indicates the absence of N protein.

A total of 22 serum samples from patients with COVID-19 (2 mild, 10 moderate, and 10 severe) were used to longitudinally evaluate IL-6 enhancement at Days 1, 5, and 10 after diagnosis, which corresponded to 3, 7, and 12 days after the onset of symptoms, respectively (Figure 2). The number of mild cases (2) was limited, as hospital stays of mild cases were shorter than the stays of other cases. IL-6 levels in the culture supernatant of K-ML2 cells after the addition of serum samples without the N protein were considered the baseline. In the presence of the N protein, mild cases showed accelerated IL-6 production up to Day 5, after which, IL-6 production plateaued until Day 10.

Among the 10 moderate cases, 7 showed rising trends from Day 1 to Day 10 with N protein stimulation. A single case showed enhancement on Day 5 and stopped rising afterward, and two cases did not show any enhancement. However, a single case without stimulation showed increased enhancement from Day 5 to Day 10 (24.16 pg/mL to 495.77 pg/mL).

Among the 10 severe cases, 6 showed daily increases in IL-6 enhancement in the presence of N protein, 2 cases showed a cessation in IL-6 enhancement after Day 5, and the remaining 2 cases did not show any evidence of enhancement. Interestingly, a single case showed enhancement from Day 1 to Day 10 (205.42 pg/mL to 487.35 pg/mL) in response to N protein stimulation, while levels declined after Day 5 in the absence of stimulation. Indeed, this case showed a persistently high level of IL-6 in serum, and the patient’s hospital stay was longer (27 days) than the stays of other patients. The findings indicated that, in longitudinal observations, IL-6 production increased significantly with N protein stimulation in most of the mild, moderate, and severe cases (*p* < 0.001, Wilcoxon signed rank test) (Figure 2).

Among the 10 severe cases, 4 deaths were recorded. In the longitudinal study, an increase in N protein-induced IL-6 production in the Day 1 to Day 5 samples from all four of the deceased patients was noted. An increase in IL-6 was also observed in Day 10 samples, except in one case (Figure 2). From the hospital information system, we found that all four cases had high serum IL-6 levels (>33 pg/mL). The fatalities were recorded in elderly patients with comorbidities, such as diabetes, hypertension, and dementia. It is possible that the dysregulated continual production of IL-6 led to the development of various diseases with increasing severity, resulting in fatal outcomes.

As shown in Table 2, the IL-6 levels in serum from severe cases were higher than those of mild and moderate cases, and this difference was statistically significant (*p* < 0.001). We counted the number of cases with serum IL-6 levels > 7 pg/mL, which is the upper limit of the normal range (<7 pg/mL), and >126 pg/mL in the culture supernatant with N protein stimulation (see Table 3). There was a trend toward increases in IL-6 levels with increasing disease severity; the proportions of patients with serum IL-6 levels > 7 pg/mL and N protein that induced IL-6 production in K-ML2 cells over 126 pg/mL were 0%, 16%, and 24% for mild, moderate, and severe cases, respectively. On the other hand, the proportions of patients with serum IL-6 in the normal range (levels < 7 pg/mL) and N protein that induced IL-6 production at levels < 126 pg/mL were 74%, 40%, and 6% for mild, moderate, and severe cases, respectively (Table 4). The results showed that the IL-6-enhancing activity of myeloid cells is at least partly related to the high IL-6 levels in serum from severely ill patients.

### 4.6. Comparison between Anti-N Antibodies and Induced IL-6 Levels: A Longitudinal Analysis of Anti-N Antibodies

**Figure 3:**
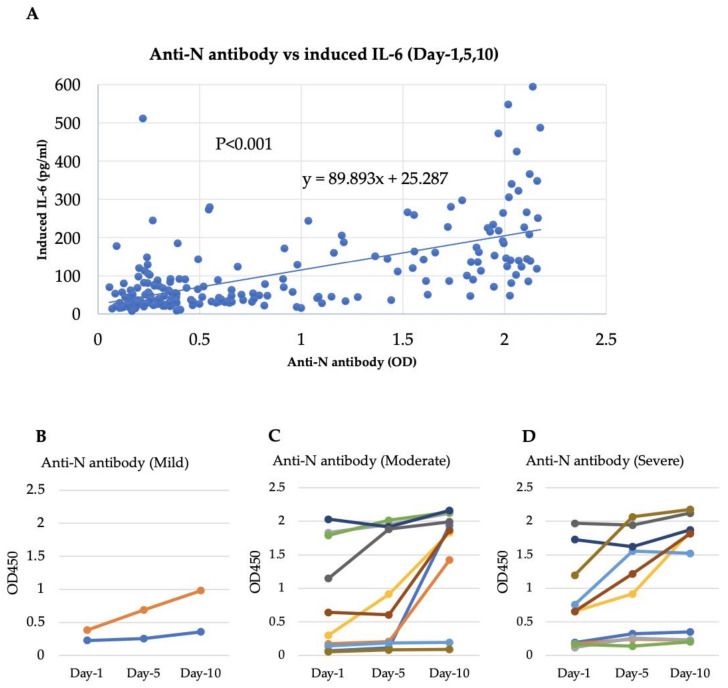
Comparison between heat-inactivated anti-N antibodies and induced IL-6 levels: a longitudinal analysis of anti-N antibodies. The position of each dot along the *x*-axis represents the OD value obtained via ELISA for the anti-N antibody, and the position on the *y*-axis indicates N protein-induced IL-6 levels (**A**). The correlation between antibody titers and induced IL-6 levels is statistically significant (R = 0.61, *p* < 0.001), as shown by Spearman’s test. A longitudinal analysis of anti-N antibodies was performed to monitor the daily enrichment of antibody concentrations in mild (**B**), moderate (**C**), and severe (**D**) cases. Different colored lines indicate different patients.

We previously showed that the anti-N antibody augments N protein-induced IL-6 production in K-ML-2 cells [42]. In the present study, the levels of anti-N antibodies were measured using an in-house ELISA system to clarify whether they were involved in the enhancement of IL-6 caused by serum and the N protein. A scatter plot analysis showed that sera with higher concentrations of anti-N antibodies had higher levels of IL-6 induction caused by the N protein (*p* < 0.001) (Figure 3A). The correlation remained statistically significant after dividing samples into Day 1 (*p* < 0.001), Day 5 (*p* < 0.001), and Day 10 (*p* < 0.001) samples (Figure S3). Levels of IL-6 induction were higher in Day 10 samples (Figure 2), and the anti-N antibody concentration was also elevated in most cases on Day 10 (Figure 3B–D). In the longitudinal analysis, an increase in the concentration of antibodies was observed in many severe and moderate cases but not in mild cases, although the number of mild cases with longitudinal data was limited in the present study (Figure 3B–D). These results showed that the anti-N antibodies in COVID-19 patient sera enhanced the IL-6 production that was induced by the N protein, especially in severe cases.

### 4.7. Lack of Correlation between Epitope Ep9 in N Protein and IL-6-Inducing Capability

**Figure 4:**
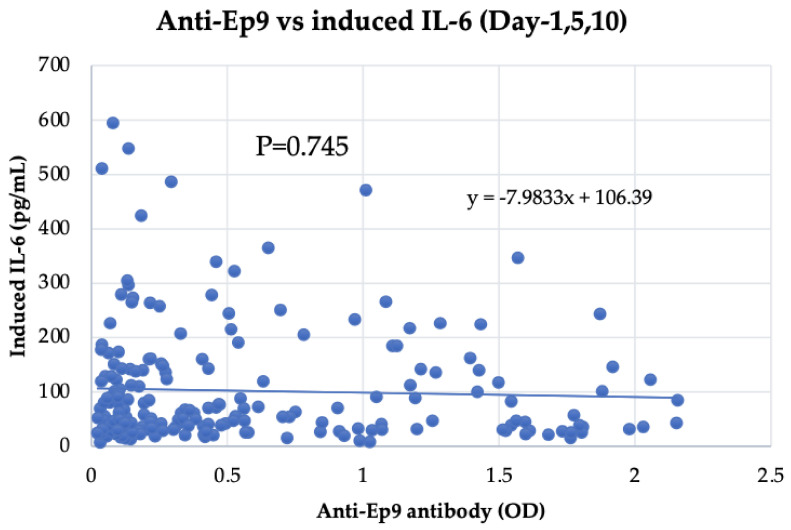
Comparison between the anti-Ep9 epitope antibody and N protein-induced IL-6 levels. Each dot’s position along the x-axis represents the OD value obtained from the ELISA analysis of the Ep9 epitope. The position on the y-axis shows N protein-induced IL-6 levels. No correlation was observed between the epitope and the induced IL-6 levels (R = 0.02, *p* = 0.745) using Spearman’s test.

It was previously reported that antibodies against a 21-amino-acid epitope at position 152–172 of the N protein, called Ep9, were correlated with COVID-19 severity [43]. Therefore, we measured levels of anti-Ep9 epitope antibodies using an in-house ELISA system to clarify the correlation between induced IL-6 levels and anti-Ep9 antibodies. No correlation was observed between the anti-Ep9 epitope antibody and the IL-6 levels induced by serum and N protein (R = 0.02; *p* = 0.745, Spearman’s test) (Figure 4). Taken together with the results shown in Figure 3, the findings suggest that the enhancement of IL-6 production was caused by anti-N antibody, which recognizes epitopes other than the Ep9 epitope.

## 5. Discussion

In the present study, we re-evaluated the correlation between COVID-19 severity and the enhancement of IL-6 production caused by myeloid cells in the presence of the N protein. Many of the serum samples collected from severe cases at 3 days after symptom onset enhanced IL-6 production in the K-ML2 cell line in the presence of the N protein, while sera from most mild cases did not. A longitudinal study with sampling at 3, 7, and 12 days after symptom onset showed that levels of IL-6 production increased daily, suggesting that anti-N antibody in patient serum plays a crucial role in increasing IL-6 production when the N protein is present. Indeed, we observed an increase in anti-N protein antibodies from longitudinally collected sera and a significant and positive correlation between the antibody concentration and the IL-6 levels that were induced by the patient sera in the presence of the N protein. These results, together with our previous finding that healthy donor sera failed to enhance IL-6 production, indicate that the anti-N antibody in the patient sera enhances IL-6 production via myeloid cells in the presence of N protein produced by infected cells, which may aggravate the disease. Several studies have shown that severely infected patients have higher anti-N antibody titers [59,60,61,62]. Regarding the biochemical and hematological parameters of our study population, we observed significant increases in IL-6, C-reactive protein, D-dimer, ferritin, creatinine, procalcitonin, hs-TnI, NT-proBNP, leukocytes, and NLR and significant decreases in hemoglobin, hematocrit, and albumin values in severe cases.

The N protein can induce the production of IL-6 via the activation of N-mediated NLRP3 [41]. We previously showed that the N protein produced in infected epithelial cells can induce IL-6 production via myeloid cells and macrophages without direct infection with SARS-CoV-2 [42]. In that study, we reported a statistically significant difference in induced IL-6 levels between patient groups categorized by disease severity. However, despite using the same system as before, the differences observed did not reach statistical significance, although a similar trend was evident (Figure 1). In the previous study, the sampling date from the onset of the disease was not controlled, and severe cases admitted to the tertiary hospital ICU were already at the late stage of the disease. In contrast, patient sera in the present study were collected at 3 days after onset for both mild and severe cases. Since the levels of IL-6, which are induced by myeloid cells from patient sera in the presence of the N protein, increased daily, significant differences could be observed if we compared these IL-6 levels at times when the patients’ conditions deteriorated. Nevertheless, when we considered the number of cases with apparently elevated IL-6 levels in both culture supernatants of K-ML2 cells in the presence of the N protein and patient sera, we observed statistically significant differences between mild, moderate, and severe cases, with higher levels of IL-6 in severe cases (Table 3). Therefore, these findings corroborate, at least in part, our previous findings.

Serum IL-6 levels have been reported to be well suited for use as a biomarker for predicting disease course [63,64,65], and this is corroborated by our study (Table 2 and Figure 3A). Patient sera with high levels of IL-6 also tended to induce high levels of IL-6 in the culture supernatant of myeloid cells in the presence of N protein, even after heat inactivation (Table 4). However, sera from several cases with elevated serum IL-6 levels did not show an increase in IL-6 induction via K-ML2 cells. Several factors could be involved in this phenomenon; for example, certain inhibitory factors in patient sera could impede IL-6 induction via K-ML2 cells, and genetic variations among individuals might also affect the levels of such inhibitory factors. It is also possible that pre-existing immunological diseases, such as rheumatoid arthritis or co-infection with other pathogens, could be involved. It should be noted here that the heat-inactivated sera from severe cases enhanced the production of higher IL-6 from myeloid cells, even in the absence of the N protein (Figure 2).

Our previous study [42] did not show a correlation between anti-N antibodies and IL-6 induction from myeloid cells. However, in the present study, we observed a significant positive correlation between the anti-N antibody and IL-6 levels induced by patient sera in the presence of the N protein (Figure 3A). Several factors can affect the dynamics of anti-N antibody concentration over time, such as early immune responses, virus replication and antigen load, peak antibody response, and antibody decline depending on infection severity and long-term immunity, which can trigger a robust antibody response from persistent memory B cells and T cells [66]. It is believed that IL-6 enhancement via patient sera that is still observable after recovery is related to the existence of anti-N antibodies, even though the N protein itself has disappeared. In longitudinal observations of anti-N antibodies, elevated antibody concentrations have been observed in severe cases [67]. These elevated antibody concentrations may be due to high viral loads; prolonged infections compared with mild cases; severe tissue damage; and dysregulated immune responses leading to a cytokine storm that can, in turn, result in an increase in the production of antibodies [68,69,70]. By using a highly sensitive microbead-based immunoassay, the ACTIV-3/TICO Study Group previously reported that elevated N protein levels are related to disease severity [71]. Unfortunately, we did not determine the levels of N protein in the patients’ sera in the present study.

Previously, Sen et al. demonstrated the presence of anti-Ep9 antibodies in patients with severe COVID-19 with diabetes or hypertension or who were aged >50 years [43]. In the present study, we did not observe a significant correlation between levels of anti-Ep9 antibodies and those of IL-6 induced in the presence of the N protein (Figure 4). The lack of consistency between antibody levels against the Ep9 epitope and the IL-6-inducing capability of patient sera could be attributed to several factors, including the genetic variability of individuals, host factors, the influence of multiple epitopes or factors other than Ep9, and diverse immune responses among individuals. IL-6 induction might be influenced by other inflammatory mediators and, hence, the relationship between Ep9 epitope antibodies and IL-6 induction may be obscured.

The present study has several limitations. Being a single-center study with a relatively small sample size, its findings may have limited generalizability. Additionally, potential confounding factors, including variations in treatment regimens, concomitant medications, comorbidities, and the retrospective study design, also contribute to the study’s limitations. The possibility that immune complexes other than SARS-CoV-2 infection were involved in the production of IL-6 could not be completely ruled out in the present study, although the statistically significant association between levels of anti-N antibody and induced IL-6 strongly supports our hypothesis.

It is not clear whether the finding that secondary infection resulted in more severe outcomes than the primary infection in a veteran cohort [72] can be generalized to a younger population. If the anti-S antibody cannot neutralize the secondary infection due to the decay of antibodies and/or escape the mutation of newly emerged variants such as BA.2.86 [73], the acquired anti-N antibody could be one of the causes of IL-6 induction [74]. Higher cytokine levels can promote a state of hyperinflammation that is harmful to host cells [75]. Recently, post-acute sequelae of COVID-19, or PASC, have become a global problem [76,77], and the frequency of PASC has not been observed to decrease in re-infected groups. In fact, the frequency of PASC was higher in re-infected groups after omicron infection [78]. Woodruff et al. stratified PASC patients into an inflammatory type with high serum IL-6 and non-inflammatory types and showed that the levels of 12 blood biomarkers could predict inflammatory PASC [79]. Orban et al. reported that PASC patients with neurological symptoms had detectable anti-N IgG antibodies over a year post-onset, while those without neurological symptoms or unexposed healthy controls did not [80]. The elevated frequency of PASC in re-infected patients likely occurs because IL-6 has a senescence-associated secretory phenotype (SASP), which causes prolonged inflammation [81].

## 6. Conclusions

Based on COVID-19 patient blood biomarkers and IL-6 production, this retrospective study supports the hypothesis that elevated levels of different biomarkers and IL-6 may reflect disease severity and clinical outcomes. IL-6 can be used as an early indicator of cytokine storms [65], which are directly associated with acute respiratory distress syndrome (ARDS) and mortality. Anti-N antibody plays a crucial role in the pathogenesis of severe COVID-19 cases through the enhancement of IL-6 production from macrophages in the presence of the N protein. The findings of this study may also have implications for the development of therapeutics and vaccines for COVID-19, as targeting the N protein may be a potential strategy for controlling the immune response and reducing inflammation in COVID-19 patients. For example, the small molecular compound that inhibits the signaling of NLRP3 induced by the N protein could be a candidate. Our data also strongly support the current vaccine strategy, which targets the S protein only.

## Figures and Tables

**Table 1:** Demographics and clinical characteristics of COVID-19 patients.

Characteristics		Mild, %	Moderate, %	Severe, %	Total, %	*p*-Value
		(*n* = 66)	(*n* = 46)	(*n* = 18)	(*n* = 130)	
Age—median year (IQR)		43 (22–58)	68 (51–75)	76 (61–81)	57 (31–72)	<0.001
Male		45 (68.2)	22 (47.8)	11 (61.1)	78 (60)	0.182
**Baseline symptoms**						
Fever		60 (90.9)	38 (82.6)	18 (100)	116 (89.2)	0.106
Cough		38 (57.6)	30 (65.2)	15 (83.3)	83 (63.8)	0.126
Runny nose		13 (19.7)	7 (15.2)	1 (5.6)	21 (16.2)	0.367
Shortness of breath		0	15 (32.6)	15 (83.3)	30 (23.1)	<0.001
Nausea or vomiting		13 (19.7)	9 (19.6)	1 (5.6)	23 (17.7)	0.374
Diarrhea		2 (3)	2 (4.3)	0	4 (3.1)	0.844
Fatigue		18 (27.3)	17 (37)	4 (22.2)	39 (30)	0.419
Headache		14 (21.2)	6 (13)	1 (5.6)	21 (16.2)	0.218
Chest pain		4 (6.1)	8 (17.4)	1 (5.6)	13 (10)	0.119
Abdominal pain		1 (1.5)	1 (2.2)	0	2 (1.5)	1
Myalgia or arthralgia		9 (13.6)	6 (13)	3 (16.7)	18 (13.8)	1
**Treatment**						
Antiviral treatment						
	Remdesivir	3 (4.5)	22 (47.8)	13 (72.2)	38 (29.2)	<0.001
	Molnupiravir	9 (13.6)	4 (8.7)	0	13 (10)	0.221
	Paxlovid	4 (6.1)	2 (4.3)	0	6 (4.6)	0.659
	Acyclovir	1 (1.5)	1 (2.2)	0	2 (1.5)	1
Antibiotic treatment		31 (47)	39 (84.8)	18 (100)	88 (67.7)	<0.001
No antiviral/antibiotic treatment		26 (39.4)	0	0	26 (20)	<0.001
Convalescent plasma therapy		0	0	3 (16.7)	3 (2.3)	0.002
Oxygen support		0	17 (37)	18 (100)	35 (26.9)	<0.001
High-flow nasal cannula		0	0	15 (83.3)	15 (11.5)	<0.001
Mechanical ventilation		0	0	3 (16.7)	3 (2.3)	0.002
Vaccinated		49 (74.2)	45 (97.8)	18 (100)	112 (86.2)	<0.001
**Clinical outcome**						
Outpatients		35 (53)	4 (8.7)	0	39 (30)	<0.001
Inpatients		31 (47)	42 (91.3)	18 (100)	91 (70)	<0.001
Hospital stays (days)—median (IQR)		2 (2–3)	5 (2–8)	10 (6–18)	4 (2–8)	<0.001
Death		0	0	11 (61.1)	11 (8.5)	<0.001

*p*-values were calculated using Pearson’s chi-square test, except for those for age and length of hospital stay, which were calculated using the Kruskal–Wallis H-test. IQR: interquartile range.

**Table 2:** Biochemical and hematological parameters by disease severity.

Laboratory Parameters	Mild		Moderate		Severe		*p*-Value
Blood Routine Biomarkers	Median (IQR)	N	Median (IQR)	N	Median (IQR)	N	
White blood cells, ×10^9^/L	6.68 (5.26–8.47)	66	8.28 (6.40–9.99)	46	11.53 (8.47–16.86)	18	<0.001
Neutrophils, ×10^9^/L	4.44 (3.32–5.70)	66	6.28 (4.53–8.09)	46	10.45 (7.41–15.32)	18	<0.001
Lymphocytes, ×10^9^/L	1.69 (1.21–2.25)	66	1.35 (1.01–1.80)	46	1.11 (0.86–1.67)	18	0.04
Hemoglobin, g/dL	12.95 (12–13.90)	66	11.5 (10.05–12.58)	46	10.2 (9.65–11.3)	18	<0.001
Platelet, ×10^9^/L	220 (177.75–284.50)	66	191 (160–249.50)	46	205.5 (156.25–256.75)	18	0.185
Hematocrit, %	38.7 (35.85–41.4)	66	33.15 (29.08–38.6)	46	28.85 (26.7–30.75)	18	<0.001
Neutrophil-to-lymphocyte ratio (NLR)	2.66 (1.84–4.03)	66	4.67 (2.92–6.42)	46	7.47 (6.14–15.62)	18	<0.001
**Inflammatory biomarkers**							
C-reactive protein, mg/dL	0.68 (0.33–1.22)	66	3.88 (1.79–5.52)	46	13.25 (6.53–19.50)	18	<0.001
Ferritin, µg/L	97 (60–119.25)	12	342.50 (143.25–702)	16	961 (271–3,037)	13	<0.001
Procalcitonin, ng/mL	0.055 (0.05–0.07)	10	0.15 (0.08–6.82)	23	3.39 (1.44–8.13)	17	0.001
Interleukin-6, pg/mL	2.87 (1.71–12.95)	66	10.74 (3.87–19.98)	45	108.65 (42.17–231.62)	18	<0.001
**Biochemistry biomarkers**							
Creatinine, mg/dL	0.88 (0.79–1.09)	53	1.25 (1.03–1.95)	43	2.15 (1.50–2.65)	17	<0.001
ALT/SGPT, U/L	27 (22–36)	37	34.5 (20.75–49.25)	40	35 (24–41)	17	0.346
AST/SGOT, U/L	25 (22.50–37)	23	40 (24.50–53.50)	35	54.5 (38.50–96.25)	16	0.002
Albumin, g/dL	3.60 (3.35–4)	19	3.10 (2.75–3.30)	31	2.60 (2.40–2.60)	15	<0.001
Coagulation biomarker							
D-dimer, ng/mL	317.50 (231.25–492.50)	34	930 (374.5–1708.5)	40	3929.5 (1960–12,858.25)	18	<0.001
**Cardiac biomarkers**							
High-sensitivity troponin I, ng/L	7.70 (5.55–32.68)	8	32.50 (8.95–112.48)	26	113 (37–594)	17	0.01
NT-proBNP, pg/mL	13 (11.50–2165)	3	426 (118–4776)	21	8898 (972–30,000)	17	0.005

*p*-values were calculated using the non-parametric Kruskal–Wallis H-test. Missing data were not included in the analysis. N: number of samples, IQR: interquartile range, ALT/SGPT: alanine aminotransferase/serum glutamic pyruvic transaminase, AST/SGOT: aspartate aminotransferase/serum glutamic–oxaloacetic transaminase, hs-TnI: high-sensitivity troponin I, NT-proBNP: N-terminal pro-b-type natriuretic peptide.

**Table 3:** Number of Day 1 samples inducing IL-6 to levels above 126 pg/mL in the presence of N protein.

IL-6	Mild Cases (%)	Moderate Cases (%)	Severe Cases (%)
>126 pg/mL	4 (6.1)	13 (28.3)	5 (27.8)
<126 pg/mL	62 (93.9)	33 (71.7)	13 (72.2)
Total	66 (100)	46 (100)	18 (100)

Note: The cut-off value of IL-6, 126 pg/mL, was determined as the 95th percentile of IL-6 levels in the absence of N protein in Day 1 samples.

**Table 4:** IL-6 levels in patient sera and in cell cultures.

		Serum IL-6		
	IL-6 Induction	<7 pg/mL	>7 pg/mL	
Category	with N Protein	Cases (%)	Cases (%)	Cases
Mild	<126 pg/mL	42 (74)	12 (21)	57
	>126 pg/mL	3 (5)	0 (0)	
Moderate	<126 pg/mL	17 (40)	15 (35)	43
	>126 pg/mL	4 (9)	7 (16)	
Severe	<126 pg/mL	1 (6)	11 (65)	17
	>126 pg/mL	1 (6)	4 (24)	

## Data Availability

All data and materials used and/or analyzed as part of this study are included in the published article.

## Supplementary Materials

The following supporting information can be downloaded at https://www.mdpi.com/article/10.3390/v15102018/s1, Figure S1: Biochemical and hematological parameters at Day 1 according to disease severity (mild, moderate, and severe); Figure S2: IL-6 induction with COVID-19 serum samples collected on Day 5 and Day 10; Figure S3: Comparison between anti-N antibodies and N-protein-induced IL-6 levels.

## References

[1] Huang C., Wang Y., Li X., Ren L., Zhao J., Hu Y., Zhang L., Fan G., Xu J., Gu X. (2020). Clinical features of patients infected with 2019 novel coronavirus in Wuhan, China. Lancet.

[2] Ciaccio M., Agnello L. (2020). Biochemical biomarkers alterations in Coronavirus Disease 2019 (COVID-19). Diagnosis.

[3] Sahin A.R., Erdogan A., Agaoglu P.M., Dineri Y., Cakirci A.Y., Senel M.E., Okyay R.A., Tasdogan A.M. (2020). 2019 novel coronavirus (COVID-19) outbreak: A review of the current literature. EJMO.

[4] Satarker S., Nampoothiri M. (2020). Structural Proteins in Severe Acute Respiratory Syndrome Coronavirus-2. Arch. Med. Res..

[5] Romano M., Ruggiero A., Squeglia F., Maga G., Berisio R. (2020). A Structural View of SARS-CoV-2 RNA Replication Machinery: RNA Synthesis, Proofreading and Final Capping. Cells.

[6] Wu A., Peng Y., Huang B., Ding X., Wang X., Niu P., Meng J., Zhu Z., Zhang Z., Wang J. (2020). Genome Composition and Divergence of the Novel Coronavirus (2019-nCoV) Originating in China. Cell Host Microbe.

[7] Lu R., Zhao X., Li J., Niu P., Yang B., Wu H., Wang W., Song H., Huang B., Zhu N. (2020). Genomic characterisation and epidemiology of 2019 novel coronavirus: Implications for virus origins and receptor binding. Lancet.

[8] Zhang Z., Nomura N., Muramoto Y., Ekimoto T., Uemura T., Liu K., Yui M., Kono N., Aoki J., Ikeguchi M. (2022). Structure of SARS-CoV-2 membrane protein essential for virus assembly. Nat. Commun..

[9] McBride R., Fielding B.C. (2012). The role of severe acute respiratory syndrome (SARS)-coronavirus accessory proteins in virus pathogenesis. Viruses.

[10] Driggin E., Madhavan M.V., Bikdeli B., Chuich T., Laracy J., Biondi-Zoccai G., Brown T.S., Der Nigoghossian C., Zidar D.A., Haythe J. (2020). Cardiovascular Considerations for Patients, Health Care Workers, and Health Systems during the COVID-19 Pandemic. J. Am. Coll. Cardiol..

[11] Xiao F., Tang M., Zheng X., Liu Y., Li X., Shan H. (2020). Evidence for Gastrointestinal Infection of SARS-CoV-2. Gastroenterology.

[12] Medetalibeyoglu A., Catma Y., Senkal N., Ormeci A., Cavus B., Kose M., Bayramlar O.F., Yildiz G., Akyuz F., Kaymakoglu S. (2020). The effect of liver test abnormalities on the prognosis of COVID-19. Ann. Hepatol..

[13] Adapa S., Chenna A., Balla M., Merugu G.P., Koduri N.M., Daggubati S.R., Gayam V., Naramala S., Konala V.M. (2020). COVID-19 Pandemic Causing Acute Kidney Injury and Impact on Patients with Chronic Kidney Disease and Renal Transplantation. J. Clin. Med. Res..

[14] Ellul M.A., Benjamin L., Singh B., Lant S., Michael B.D., Easton A., Kneen R., Defres S., Sejvar J., Solomon T. (2020). Neurological associations of COVID-19. Lancet Neurol..

[15] Broxmeyer H.E., Parker G.C. (2020). Impact of COVID-19 and Future Emerging Viruses on Hematopoietic Cell Transplantation and Other Cellular Therapies. Stem. Cells Dev..

[16] Chen G., Wu D., Guo W., Cao Y., Huang D., Wang H., Wang T., Zhang X., Chen H., Yu H. (2020). Clinical and immunological features of severe and moderate coronavirus disease 2019. J. Clin. Investig..

[17] Schultze J.L., Aschenbrenner A.C. (2021). COVID-19 and the human innate immune system. Cell.

[18] Tay M.Z., Poh C.M., Renia L., MacAry P.A., Ng L.F.P. (2020). The trinity of COVID-19: Immunity, inflammation and intervention. Nat. Rev. Immunol..

[19] Burke H., Freeman A., Cellura D.C., Stuart B.L., Brendish N.J., Poole S., Borca F., Phan H.T.T., Sheard N., Williams S. (2020). Inflammatory phenotyping predicts clinical outcome in COVID-19. Respir. Res..

[20] Del Valle D.M., Kim-Schulze S., Huang H.H., Beckmann N.D., Nirenberg S., Wang B., Lavin Y., Swartz T.H., Madduri D., Stock A. (2020). An inflammatory cytokine signature predicts COVID-19 severity and survival. Nat. Med..

[21] Manik M., Singh R.K. (2022). Role of toll-like receptors in modulation of cytokine storm signaling in SARS-CoV-2-induced COVID-19. J. Med. Virol..

[22] Chen T., Lin Y.X., Zha Y., Sun Y., Tian J., Yang Z., Lin S.W., Yu F., Chen Z.S., Kuang B.H. (2021). A Low-Producing Haplotype of Interleukin-6 Disrupting CTCF Binding Is Protective against Severe COVID-19. mBio.

[23] Elahi R., Karami P., Heidary A.H., Esmaeilzadeh A. (2022). An updated overview of recent advances, challenges, and clinical considerations of IL-6 signaling blockade in severe coronavirus disease 2019 (COVID-19). Int. Immunopharmacol..

[24] Ashrafzadeh-Kian S., Campbell M.R., Jara Aguirre J.C., Walsh J., Kumanovics A., Jenkinson G., Rinaldo P., Snyder M.R., Algeciras-Schimnich A. (2022). Role of immune mediators in predicting hospitalization of SARS-CoV-2 positive patients. Cytokine.

[25] Jose R.J., Manuel A. (2020). COVID-19 cytokine storm: The interplay between inflammation and coagulation. Lancet Respir. Med..

[26] Darif D., Hammi I., Kihel A., El Idrissi Saik I., Guessous F., Akarid K. (2021). The pro-inflammatory cytokines in COVID-19 pathogenesis: What goes wrong?. Microb. Pathog..

[27] Hirano T. (2021). IL-6 in inflammation, autoimmunity and cancer. Int. Immunol..

[28] Tanaka T., Narazaki M., Kishimoto T. (2014). IL-6 in inflammation, immunity, and disease. Cold Spring Harb. Perspect. Biol..

[29] Hirano T. (2010). Interleukin 6 in autoimmune and inflammatory diseases: A personal memoir. Proc. Jpn. Acad. Ser. B Phys. Biol. Sci..

[30] Testing.com. Interleukin-6: OneCare Media, 2023 [updated Nov 9, 2021]. https://www.testing.com/tests/interleukin-6.

[31] Ulhaq Z.S., Soraya G.V. (2020). Interleukin-6 as a potential biomarker of COVID-19 progression. Med. Mal. Infect.

[32] Cifaldi L., Prencipe G., Caiello I., Bracaglia C., Locatelli F., De Benedetti F., Strippoli R. (2015). Inhibition of natural killer cell cytotoxicity by interleukin-6: Implications for the pathogenesis of macrophage activation syndrome. Arthritis Rheumatol..

[33] Zeng Z., Yu H., Chen H., Qi W., Chen L., Chen G., Yan W., Chen T., Ning Q., Han M. (2020). Longitudinal changes of inflammatory parameters and their correlation with disease severity and outcomes in patients with COVID-19 from Wuhan, China. Crit. Care.

[34] Fu B., Xu X., Wei H. (2020). Why tocilizumab could be an effective treatment for severe COVID-19?. J. Transl. Med..

[35] Zhao M., Lu J., Tang Y., Dai Y., Zhou J., Wu Y. (2021). Tocilizumab for treating COVID-19: A systemic review and meta-analysis of retrospective studies. Eur. J. Clin. Pharmacol..

[36] Michot J.M., Albiges L., Chaput N., Saada V., Pommeret F., Griscelli F., Balleyguier C., Besse B., Marabelle A., Netzer F. (2020). Tocilizumab, an anti-IL-6 receptor antibody, to treat COVID-19-related respiratory failure: A case report. Ann. Oncol..

[37] Galvan-Roman J.M., Rodriguez-Garcia S.C., Roy-Vallejo E., Marcos-Jimenez A., Sanchez-Alonso S., Fernandez-Diaz C., Alcaraz-Serna A., Mateu-Albero T., Rodriguez-Cortes P., Sanchez-Cerrillo I. (2021). IL-6 serum levels predict severity and response to tocilizumab in COVID-19: An observational study. J. Allergy Clin. Immunol..

[38] Shimizu J., Sasaki T., Yamanaka A., Ichihara Y., Koketsu R., Samune Y., Cruz P., Sato K., Tanga N., Yoshimura Y. (2021). The potential of COVID-19 patients’ sera to cause antibody-dependent enhancement of infection and IL-6 production. Sci. Rep..

[39] Karwaciak I., Salkowska A., Karas K., Dastych J., Ratajewski M. (2021). Nucleocapsid and Spike Proteins of the Coronavirus SARS-CoV-2 Induce IL6 in Monocytes and Macrophages-Potential Implications for Cytokine Storm Syndrome. Vaccines.

[40] Zhang X., Wu K., Wang D., Yue X., Song D., Zhu Y., Wu J. (2007). Nucleocapsid protein of SARS-CoV activates interleukin-6 expression through cellular transcription factor NF-kappaB. Virology.

[41] Pan P., Shen M., Yu Z., Ge W., Chen K., Tian M., Xiao F., Wang Z., Wang J., Jia Y. (2021). SARS-CoV-2 N protein promotes NLRP3 inflammasome activation to induce hyperinflammation. Nat. Commun..

[42] Nakayama E.E., Kubota-Koketsu R., Sasaki T., Suzuki K., Uno K., Shimizu J., Okamoto T., Matsumoto H., Matsuura H., Hashimoto S. (2022). Anti-nucleocapsid antibodies enhance the production of IL-6 induced by SARS-CoV-2 N protein. Sci. Rep..

[43] Sen S.R., Sanders E.C., Gabriel K.N., Miller B.M., Isoda H.M., Salcedo G.S., Garrido J.E., Dyer R.P., Nakajima R., Jain A. (2021). Predicting COVID-19 Severity with a Specific Nucleocapsid Antibody plus Disease Risk Factor Score. mSphere.

[44] Grasselli G., Greco M., Zanella A., Albano G., Antonelli M., Bellani G., Bonanomi E., Cabrini L., Carlesso E., Castelli G. (2020). Risk Factors Associated with Mortality among Patients with COVID-19 in Intensive Care Units in Lombardy, Italy. JAMA Intern. Med..

[45] Bwire G.M. (2020). Coronavirus: Why Men are More Vulnerable to COVID-19 Than Women?. SN Compr. Clin. Med..

[46] Zhou F., Yu T., Du R., Fan G., Liu Y., Liu Z., Xiang J., Wang Y., Song B., Gu X. (2020). Clinical course and risk factors for mortality of adult inpatients with COVID-19 in Wuhan, China: A retrospective cohort study. Lancet.

[47] Tang N., Bai H., Chen X., Gong J., Li D., Sun Z. (2020). Anticoagulant treatment is associated with decreased mortality in severe coronavirus disease 2019 patients with coagulopathy. J. Thromb. Haemost..

[48] Lippi G., Plebani M. (2020). The critical role of laboratory medicine during coronavirus disease 2019 (COVID-19) and other viral outbreaks. Clin. Chem. Lab. Med..

[49] Wu C., Chen X., Cai Y., Xia J., Zhou X., Xu S., Huang H., Zhang L., Zhou X., Du C. (2020). Risk Factors Associated with Acute Respiratory Distress Syndrome and Death in Patients with Coronavirus Disease 2019 Pneumonia in Wuhan, China. JAMA Intern. Med..

[50] Liu Y., Du X., Chen J., Jin Y., Peng L., Wang H.H.X., Luo M., Chen L., Zhao Y. (2020). Neutrophil-to-lymphocyte ratio as an independent risk factor for mortality in hospitalized patients with COVID-19. J. Infect..

[51] Toori K.U., Qureshi M.A., Chaudhry A., Safdar M.F. (2021). Neutrophil to lymphocyte ratio (NLR) in COVID-19: A cheap prognostic marker in a resource constraint setting. Pak. J. Med. Sci..

[52] Li X., Liu C., Mao Z., Xiao M., Wang L., Qi S., Zhou F. (2020). Predictive values of neutrophil-to-lymphocyte ratio on disease severity and mortality in COVID-19 patients: A systematic review and meta-analysis. Crit. Care.

[53] Yang A.P., Liu J.P., Tao W.Q., Li H.M. (2020). The diagnostic and predictive role of NLR, d-NLR and PLR in COVID-19 patients. Int. Immunopharmacol..

[54] Varga Z., Flammer A.J., Steiger P., Haberecker M., Andermatt R., Zinkernagel A.S., Mehra M.R., Schuepbach R.A., Ruschitzka F., Moch H. (2020). Endothelial cell infection and endotheliitis in COVID-19. Lancet.

[55] El Hajj S., Canabady-Rochelle L., Gaucher C. (2023). Nature-Inspired Bioactive Compounds: A Promising Approach for Ferroptosis-Linked Human Diseases?. Molecules.

[56] Hirschhorn T., Stockwell B.R. (2019). The development of the concept of ferroptosis. Free Radic. Biol. Med..

[57] Cavezzi A., Troiani E., Corrao S. (2020). COVID-19: Hemoglobin, iron, and hypoxia beyond inflammation. A narrative review. Clin. Pract..

[58] Hasan A., Rahim R., Rahman M. (2021). Alteration of biomarkers of expired and cured COVID-19 ICU patients in a tertiary care hospital. Bioresearch Commun..

[59] Batra M., Tian R., Zhang C., Clarence E., Sacher C.S., Miranda J.N., De La Fuente J.R.O., Mathew M., Green D., Patel S. (2021). Role of IgG against N-protein of SARS-CoV2 in COVID19 clinical outcomes. Sci. Rep..

[60] Sun B., Feng Y., Mo X., Zheng P., Wang Q., Li P., Peng P., Liu X., Chen Z., Huang H. (2020). Kinetics of SARS-CoV-2 specific IgM and IgG responses in COVID-19 patients. Emerg. Microbes Infect..

[61] Li K., Huang B., Wu M., Zhong A., Li L., Cai Y., Wang Z., Wu L., Zhu M., Li J. (2020). Dynamic changes in anti-SARS-CoV-2 antibodies during SARS-CoV-2 infection and recovery from COVID-19. Nat. Commun..

[62] Hashem A.M., Algaissi A., Almahboub S.A., Alfaleh M.A., Abujamel T.S., Alamri S.S., Alluhaybi K.A., Hobani H.I., AlHarbi R.H., Alsulaiman R.M. (2020). Early Humoral Response Correlates with Disease Severity and Outcomes in COVID-19 Patients. Viruses.

[63] Santa Cruz A., Mendes-Frias A., Oliveira A.I., Dias L., Matos A.R., Carvalho A., Capela C., Pedrosa J., Castro A.G., Silvestre R. (2021). Interleukin-6 Is a Biomarker for the Development of Fatal Severe Acute Respiratory Syndrome Coronavirus 2 Pneumonia. Front. Immunol..

[64] Avila-Nava A., Cortes-Telles A., Torres-Erazo D., Lopez-Romero S., Chim Ake R., Gutierrez Solis A.L. (2021). Serum IL-6: A potential biomarker of mortality among SARS-CoV-2 infected patients in Mexico. Cytokine.

[65] Lee J.H., Jang J.H., Park J.H., Jang H.J., Park C.S., Lee S., Kim S.H., Kim J.Y., Kim H.K. (2021). The role of interleukin-6 as a prognostic biomarker for predicting acute exacerbation in interstitial lung diseases. PLoS ONE.

[66] Huang A.T., Garcia-Carreras B., Hitchings M.D.T., Yang B., Katzelnick L.C., Rattigan S.M., Borgert B.A., Moreno C.A., Solomon B.D., Trimmer-Smith L. (2020). A systematic review of antibody mediated immunity to coronaviruses: Kinetics, correlates of protection, and association with severity. Nat. Commun..

[67] Gerhards C., Thiaucourt M., Kittel M., Becker C., Ast V., Hetjens M., Neumaier M., Haselmann V. (2021). Longitudinal assessment of anti-SARS-CoV-2 antibody dynamics and clinical features following convalescence from a COVID-19 infection. Int. J. Infect. Dis..

[68] Liu Y., Yan L.M., Wan L., Xiang T.X., Le A., Liu J.M., Peiris M., Poon L.L.M., Zhang W. (2020). Viral dynamics in mild and severe cases of COVID-19. Lancet Infect. Dis..

[69] Manjili R.H., Zarei M., Habibi M., Manjili M.H. (2020). COVID-19 as an Acute Inflammatory Disease. J. Immunol..

[70] Zanza C., Romenskaya T., Manetti A.C., Franceschi F., La Russa R., Bertozzi G., Maiese A., Savioli G., Volonnino G., Longhitano Y. (2022). Cytokine Storm in COVID-19: Immunopathogenesis and Therapy. Medicina.

[71] Group A.-T.S., Rogers A.J., Wentworth D., Phillips A., Shaw-Saliba K., Dewar R.L., Aggarwal N.R., Babiker A.G., Chang W., Dharan N.J. (2022). The Association of Baseline Plasma SARS-CoV-2 Nucleocapsid Antigen Level and Outcomes in Patients Hospitalized with COVID-19. Ann. Intern. Med..

[72] Bowe B., Xie Y., Al-Aly Z. (2022). Acute and postacute sequelae associated with SARS-CoV-2 reinfection. Nat. Med..

[73] Yang S., Yu Y., Jian F., Song W., Yisimayi A., Chen X., Xu Y., Wang P., Wang J., Yu L. (2023). Antigenicity and infectivity characterization of SARS-CoV-2 BA.2.86. bioRxiv.

[74] Nakayama E.E., Shioda T. (2023). SARS-CoV-2 Related Antibody-Dependent Enhancement Phenomena In Vitro and In Vivo. Microorganisms.

[75] Huang W., Li M., Luo G., Wu X., Su B., Zhao L., Zhang S., Chen X., Jia M., Zhu J. (2021). The Inflammatory Factors Associated with Disease Severity to Predict COVID-19 Progression. J. Immunol..

[76] Munipalli B., Seim L., Dawson N.L., Knight D., Dabrh A.M.A. (2022). Post-acute sequelae of COVID-19 (PASC): A meta-narrative review of pathophysiology, prevalence, and management. SN Compr. Clin. Med..

[77] Lam I.C.H., Wong C.K.H., Zhang R., Chui C.S.L., Lai F.T.T., Li X., Chan E.W.Y., Luo H., Zhang Q., Man K.K.C. (2023). Long-term post-acute sequelae of COVID-19 infection: A retrospective, multi-database cohort study in Hong Kong and the UK. EClinicalMedicine.

[78] Thaweethai T., Jolley S.E., Karlson E.W., Levitan E.B., Levy B., McComsey G.A., McCorkell L., Nadkarni G.N., Parthasarathy S., Singh U. (2023). Development of a Definition of Postacute Sequelae of SARS-CoV-2 Infection. JAMA.

[79] Woodruff M.C., Bonham K.S., Anam F.A., Walker T.A., Faliti C.E., Ishii Y., Kaminski C.Y., Ruunstrom M.C., Cooper K.R., Truong A.D. (2023). Chronic inflammation, neutrophil activity, and autoreactivity splits long COVID. Nat. Commun..

[80] Orban Z.S., Visvabharathy L., Perez Giraldo G.S., Jimenez M., Koralnik I.J. (2023). SARS-CoV-2–Specific Immune Responses in Patients with Postviral Syndrome After Suspected COVID-19. Neurol. Neuroimmunol. Neuroinflamm..

[81] Tsuji S., Minami S., Hashimoto R., Konishi Y., Suzuki T., Kondo T., Sasai M., Torii S., Ono C., Shichinohe S. (2022). SARS-CoV-2 infection triggers paracrine senescence and leads to a sustained senescence-associated inflammatory response. Nat. Aging.

