# Supplementary material for: Enhancement of IL-6 Production Induced by SARS-CoV-2 Nucleocapsid Protein and Bangladeshi COVID-19 Patients’ Sera
Source: Viruses. 2023 Sep 28;15(10):2018. doi: 10.3390/v15102018 (PMC10611338; doi:10.3390/v15102018)
Supplement: Supplementary file 1 [file viruses-15-02018-s001.zip › viruses-2595042-supplementary.pdf]

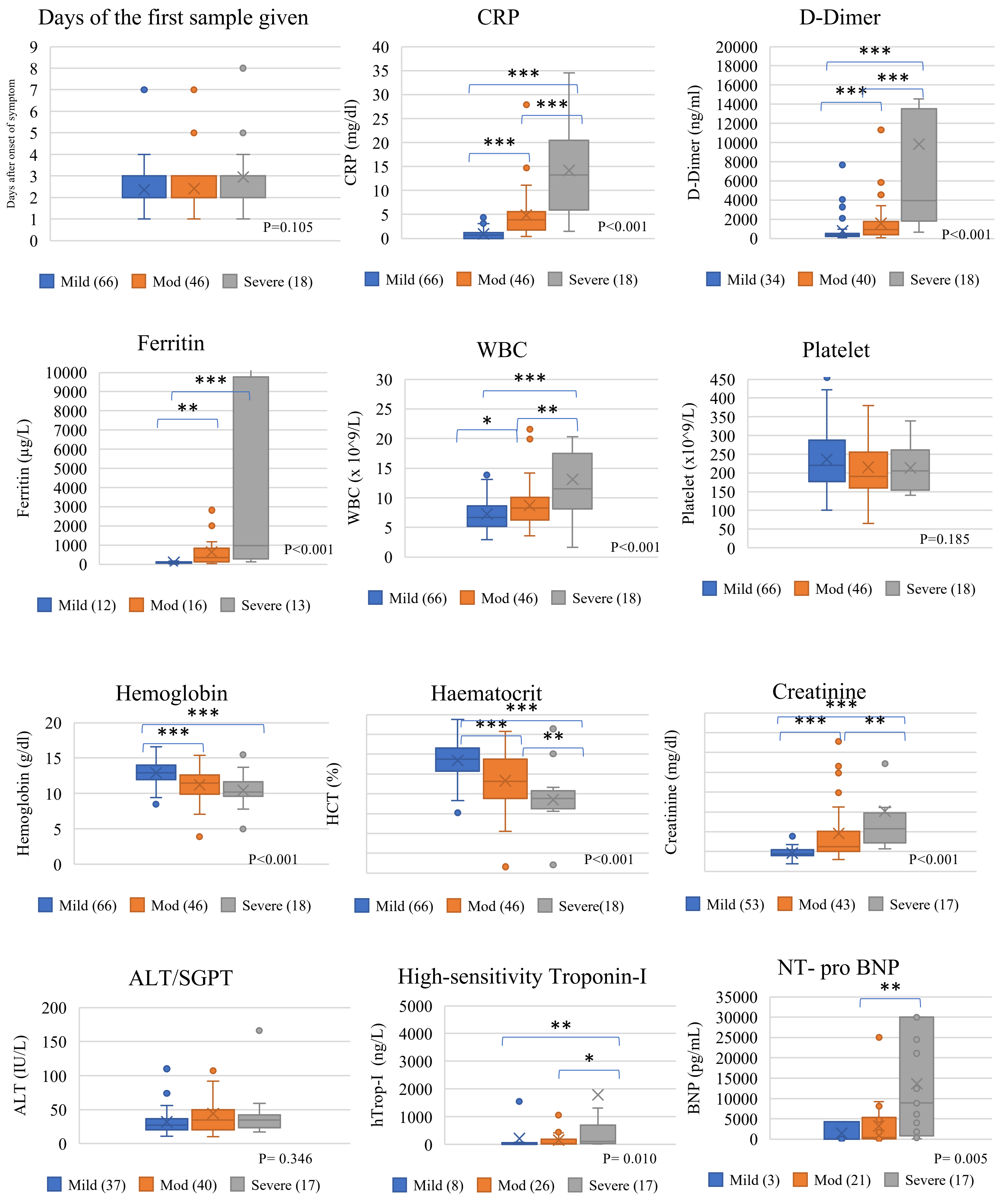
Days of the first sample given
Days after onset of symptom
P=0.105
Mild (66) Mod (46) Severe (18)
CRP
CRP (mg/dl)
P<0.001
Mild (66) Mod (46) Severe (18)
D-Dimer
D-Dimer (ng/ml)
P<0.001
Mild (34) Mod (40) Severe (18)
Ferritin
Ferritin (µg/L)
P<0.001
Mild (12) Mod (16) Severe (13)
WBC
WBC (x 10^9/L)
P<0.001
Mild (66) Mod (46) Severe (18)
Platelet
Platelet (x10^9/L)
P=0.185
Mild (66) Mod (46) Severe (18)
Hemoglobin
Hemoglobin (g/dl)
P<0.001
Mild (66) Mod (46) Severe (18)
Haematocrit
HCT (%)
P<0.001
Mild (66) Mod (46) Severe(18)
Creatinine
Creatinine (mg/dl)
P<0.001
Mild (53) Mod (43) Severe (17)
ALT/SGPT
ALT (IU/L)
P= 0.346
Mild (37) Mod (40) Severe (17)
High-sensitivity Troponin-I
hTrop-I (ng/L)
P= 0.010
Mild (8) Mod (26) Severe (17)
NT- pro BNP
BNP (pg/mL)
P= 0.005
Mild (3) Mod (21) Severe (17)

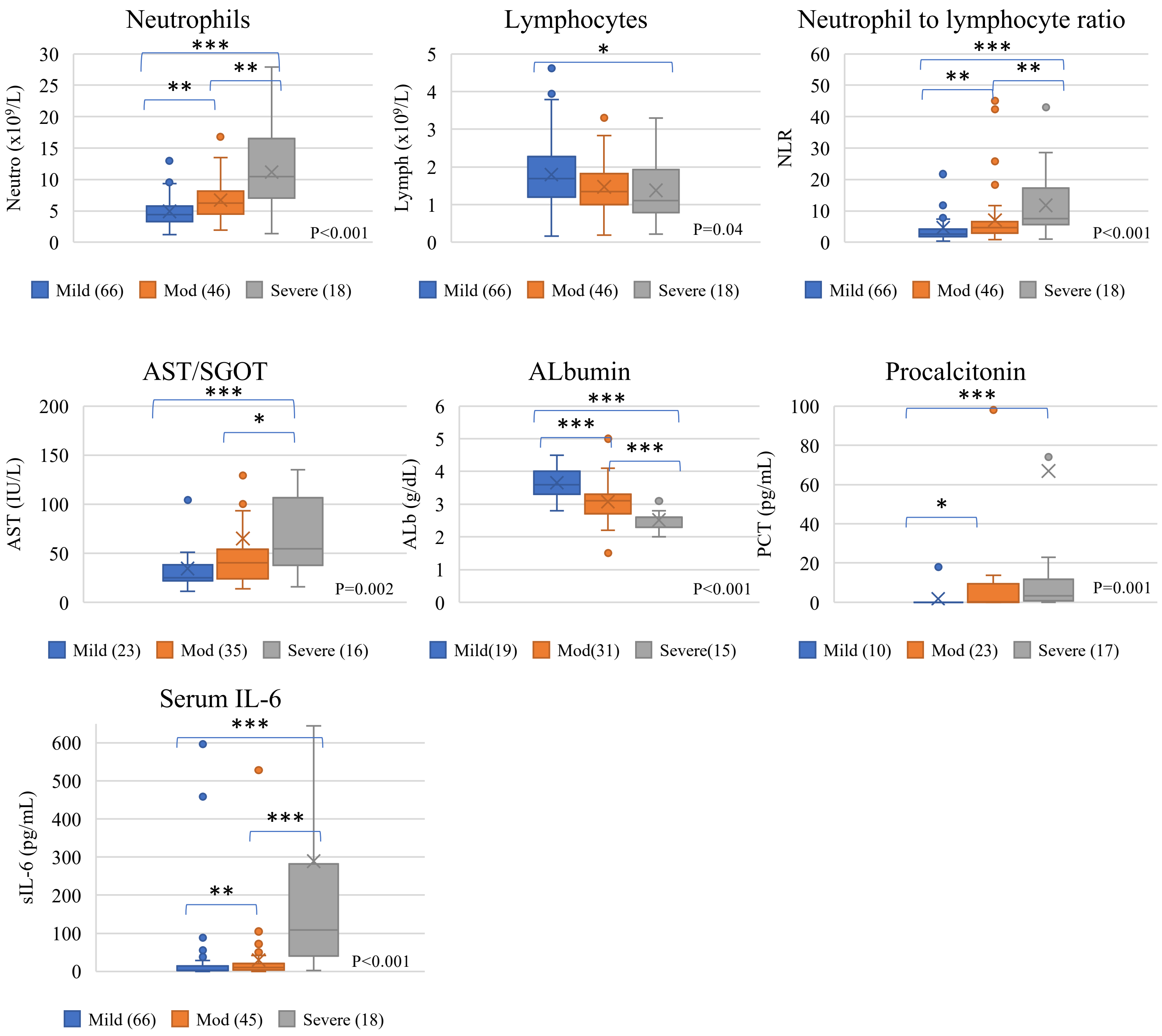

Figure S1: Biochemical and hematological parameters at Day-1 according to diseases severity (Mild, moderate and severe). The blue, orange and gray denote mild, moderate and severe cases, respectively. The center lines in the boxes, the boxes, and the vertical bars indicate the median, 25/75 percentiles, and 5/95 percentiles, respectively. P-values were calculated using Kruskal-Wallis H-test for three groups and are shown at the lower right of each panel. For pairwise comparison between Mild and Moderate, Moderate and Severe, and Mild and Severe cases, Mann-Whitney U test was used and results are shown above the brackets. Statistically significant results were indicated by asterisks (*). *, **, *** indicates P<0.05, P<0.01, P<0.001, respectively. CRP: C-reactive protein, WBC: White blood cell, ALT/SGPT: Alanine aminotransferase / serum glutamic pyruvic transaminase, AST/SGOT: Aspartate aminotransferase / serum glutamic-oxaloacetic transaminase, NT-proBNP: N-terminal pro b-type natriuretic peptide. The numbers of samples tested are noted in the legend of each panel.

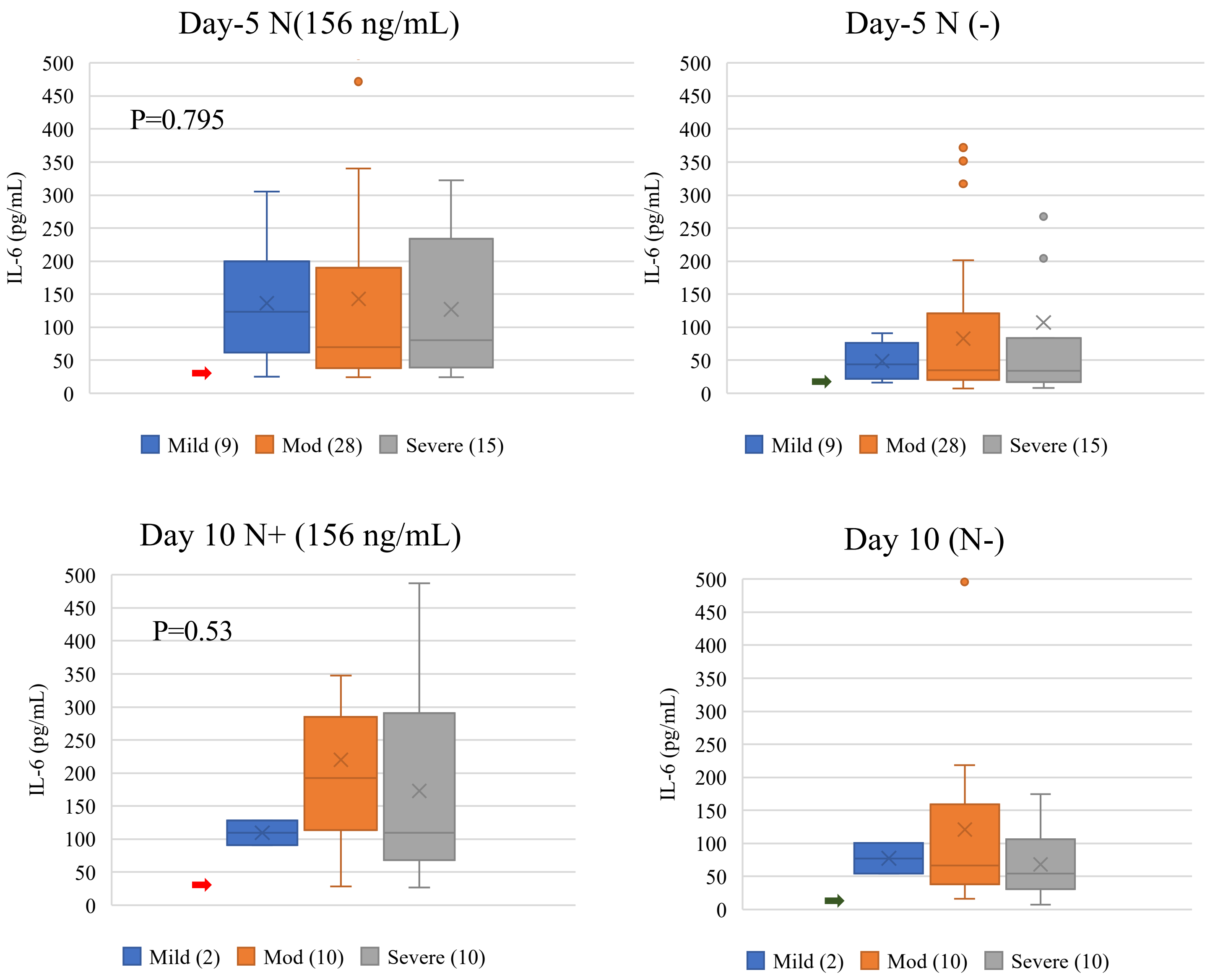

Figure S2: IL-6 induction with COVID-19 serum samples collected at Day 5 and Day 10. A total of 156 ng/mL N protein was added to K-ML2 cells to enhance IL-6 production together with 1% patients' serum. The blue, orange, and grey bars indicate mild, moderate, severe cases, respectively. The horizontal lines in the boxes, the boxes, and the vertical bars indicate the median, 25/75 percentiles, and 5/95 percentiles, respectively. The red arrow indicates IL-6 level of N protein only, and the green arrow indicates the base level of IL-6. "X" indicates the mean. The numbers of samples tested are noted in the legend of each panel. N indicates the presence of N protein, N(-) indicates the absence of N protein. The Kruskal-Wallis H-test analysis revealed that there were no significant differences among Day-5 (P=0.795) and Day-10 (P=0.53) samples.

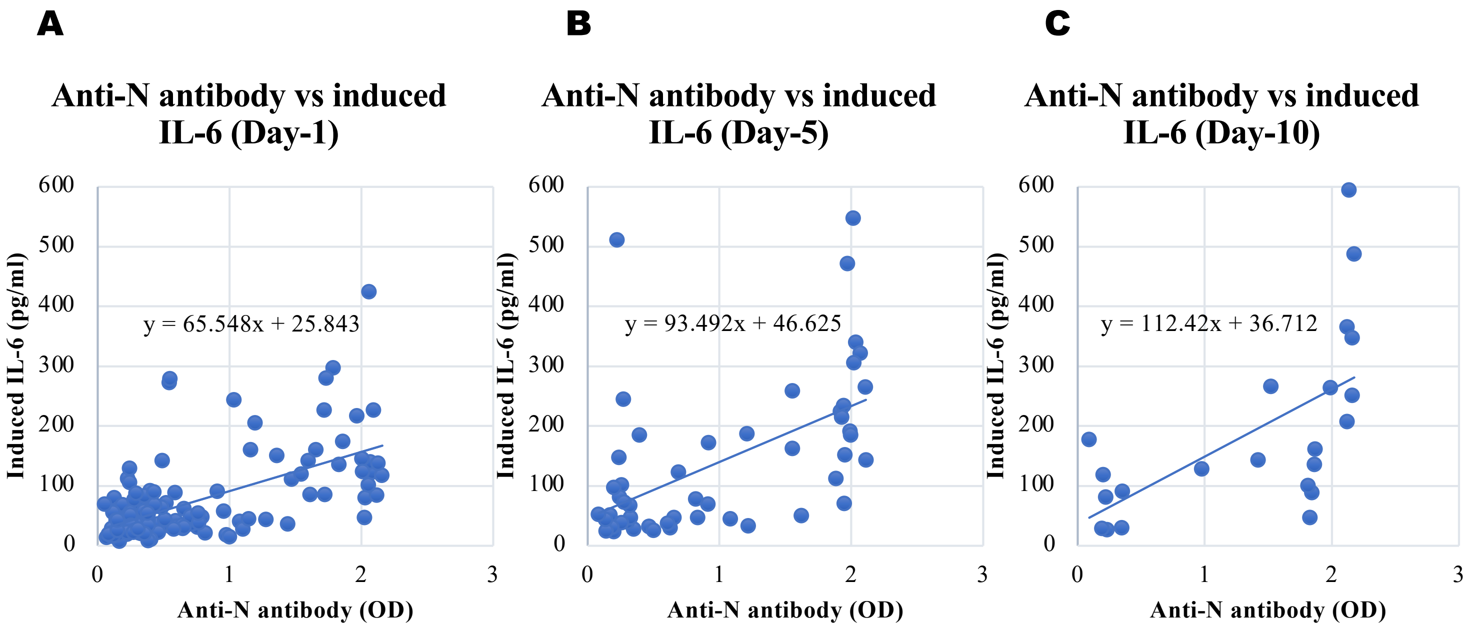

Figure S3: Comparison between anti-N antibodies and N-protein induced IL-6 levels. Each dot's position on the X-axis represents the OD value obtained by ELISA for the anti-N antibody and its position on the Y-axis indicates N protein induced IL-6 levels. The correlation between antibody titers and induced IL-6 levels was estimated to be statistically significant (Day 1: R=0.57, P<0.001; Day 5: R=0.58, P<0.001; Day 10: R=0.72, P<0.001) by Spearman's test.
